# Inflammatory-Related Clinical and Metabolic Outcomes in COVID-19 Patients

**DOI:** 10.1155/2020/2914275

**Published:** 2020-11-25

**Authors:** Maria Martinez-Urbistondo, Alberto Mora-Vargas, Esther Expósito-Palomo, Raquel Castejón, M. Jesús Citores, Silvia Rosado, Carmen de Mendoza, Isolina Baños, Ana Fernández-Cruz, Lidia Daimiel, Rodrigo San-Cristóbal, Juan Antonio Vargas, J. Alfredo Martinez

**Affiliations:** ^1^Internal Medicine Department, Hospital Universitario Puerta de Hierro Majadahonda, Madrid, Spain; ^2^Precision Nutrition Program. IMDEAFood. UAM-CSIC. Madrid, Spain; ^3^CIBERobn. Instituto Carlos III. Madrid, Spain

## Abstract

**Background:**

Severe Acute Respiratory Syndrome Coronavirus 2 (SARS-COV-2) infection elicits inflammatory manifestations that relate with a “cytokine storm.”

**Objective:**

The aim of this research was to assess the role of circulating interleukin 6 (IL-6) levels and other inflammatory markers in patients with coronavirus disease 2019 (COVID-19) on metabolic functions and accompanying clinical complications. *Patients and Methods*. A total of 165 patients diagnosed with COVID-19 pneumonia were examined for medical features and inflammatory markers such as blood IL-6, CRP, ferritin, LDH, neutrophil/lymphocyte index (NLI), D-Dimer, and Red Cell Distribution Width (RDW). Regression analyses concerning electronically collected medical data were adjusted by appropriate factors and confounding variables*. Results*. Plasma IL-6 determinations evidenced a consistent association with hospital stay days, Intensive Care Unit (ICU) admission, and mortality rates. Similar trends were found for other proinflammatory variables, where ferritin and NLI showed a remarkable value as surrogates. Hyperglycaemia and the Charlson Comorbidity Index Score were positively associated with the inflammatory response induced by the SARS-COV-2 infection. An unhealthy lifestyle such as smoking and alcoholic drinks consumption as well as excessive body adiposity influenced inflammatory-related outcomes in the screened patients.

**Conclusion:**

IL-6 together with other inflammatory biomarkers accompanied poor clinical and metabolic outcomes in COVID-19-infected patients. IL-6 may result in a suitable proxy to individually categorise patients in order to manage this infectious pandemic.

## 1. Introduction

Coronavirus disease 2019 (COVID-19) has rapidly spread around the world being declared as a pandemic by the World Health Organization (WHO) in March 2020 [1]. This situation is based on a high transmission rate, lethality, medical progression uncertainties, and the lack of satisfactory antiviral treatments [2, 3]. Thousands of COVID-19 cases have been reported encompassing a wide spectrum of symptoms [4]. The respiratory system was the most frequently affected by the Severe Acute Respiratory Syndrome Coronavirus 2 (SARS-COV-2), with patients presenting mild illness as well as more severe features such as acute respiratory distress syndrome (ARDS) requiring admission in Intensive Care Units (ICU) and specific medical attention [5]. Gastrointestinal problems such as diarrhoea or vomiting and haematological and dermatological findings have also been described in COVID-19-infected patients [6].

The average incubation period for COVID-19 is 6 to 7 days, lasting the first viremic phase from 8 to 10 days, which in most cases was followed by infection resolution [7]. However, the remaining patients progress to a second phase—called the inflammatory stage—featuring ARDS, thromboembolic events, and myocardial acute injury, which regrettably involves poor prognosis linked to an exacerbation of the immune system cascade [8].

Clinical manifestations in COVID-19 patients correlate with a phenomenon known as “cytokine storm” [9]. Thus, the major burden occurs when the host immune response to the virus is inadequate [10]. The process initiates with SARS-CoV-2 entering into cells, which is followed by an interaction with surface receptors (angiotensin-converting enzyme 2 receptor—ACE2-). Most of these receptors are located in epithelial type 2 cells from the upper respiratory tract, which leads to damage-associated molecules delivering with a subsequent activation of the innate immune system. Epithelial and endothelial cells as well as macrophages are then triggered, which release IL-6, IP-10 (interferon gamma-induced protein-10), MIG chemokine, and MCP-1 (monocyte chemoattractant protein-1) mediators and generate further inflammation with the involvement of monocytes and T cells.

Interleukin-6 (IL-6) has been identified as an essential proinflammatory molecule mediating the activation of the Janus kinase-signal transducer and transcription (JAK-STAT) pathway, which produces oxidative stress, cell proliferation, and a virus clearing default within a process called “second wave in cytokine storm” [11]. Downregulating the Suppressor of Cytokine Signalling 3 (SOCS3) may partially explain the elevated serum IL-6 levels in those patients with the most worrying symptomatology [12]. Understanding these mechanisms is especially valuable considering the proposed administration of anti-inflammatory drugs to COVID-19-infected patients [13, 14].

Mortality rates associated to COVID-19 are diverse among studies, ranging from 3% to 15% [15]. These variations are probably attributable to the different sample size, origin, diagnostic criteria, and applied tests among investigations. Several epidemiological analyses have evidenced that almost 15% of patients required ICU admission receiving mechanical ventilation and implying longer hospitalisation stays [16], which was also dependent of the baseline metabolic and clinical status [1].

The aim of this study was to evaluate the influence of the proinflammatory profile as categorised by the IL-6 levels in patients infected by SARS-COV-2, including the assessment of potential associations with other inflammatory biomarkers and the personalised impact of the inflammatory status on clinical outcomes.

## 2. Material and Methods

This investigation is a retrospective, cross-sectional, and single-centre study encompassing a cohort of 165 consecutively admitted patients to the Puerta de Hierro Hospital in Majadahonda (Madrid, Spain) between March and April 2020.

Adult subjects diagnosed with COVID-19 pneumonia according to the WHO directions were recruited. The WHO criteria were followed including cases presenting clinical signs of pneumonia (fever, cough, dyspnoea, fast breathing) plus respiratory rate > 30 breaths/min, severe respiratory distress, or SpO2 < 90% on room air [17]. Every patient had a positive PCR for SARS-COV-2 and an IL-6 measurement at admission, which was assessed following the habitual hospital laboratory operative procedures. After data collection and analysis, the participants were stratified into three groups of patients based on the interleukin measurement: patients with IL-6 between 0 and 10 pg/ml, patients with IL-6 between 10 and 100 pg/ml, and patients with IL-6 greater than 100 pg/ml [7].

At admission, study variables related to proinflammatory factors and prognosis were age, sex, IL-6 value, leukocyte counts, neutrophil/lymphocyte index (NLI), and transaminases as well as Gamma Glutamyl Transpeptidase (GGT), C-reactive Protein (CRP), lactate dehydrogenase (LDH), ferritin, total cholesterol, baseline blood glucose, D-Dimer, and Red Cell Distribution Width (RDW), which were analysed following the hospital standardised protocols. The extraction date was registered considering the first day of admission as the baseline. The outcome variables were hospital stay days, admission to the ICU, and mortality. Our alternative phrasing: Information about smoking and drinking as well as body weight and height to estimate the body mass index (kg/m^2^) were also compiled in the clinical history besides data required in the Charlson Comorbidity Index Score.

Proinflammatory markers were collected from the emergency checklist and from the admission analysis records. IL-6 quantification required ELISA (Sigma-Aldrich Human IL-6 ELISA Kit) and CBA (Cytometric Bead Array, BD Biosciences) techniques with a later flow cytometry analysis as described by the supplier. No extra blood samples were required far from those needed for the clinical practice, since all required markers were included as mandatory measurements in the local COVID-19 disease protocol.

Data collection was performed through established electronic medical records (SELENE System, Cerner Iberia, S.L.U, Madrid, Spain) with a standardised data collection form. Two senior physicians reviewed all of them for consistency. Data were anonymously used according to the authorization of the Research Ethics Committee of our hospital (PI94/20). This study complied with the guidelines involving the EU and Spanish legislation on data protection and the Declaration of Helsinki. The authors did not receive any external source of funding, but were covered by their employers.

Quantitative variables were expressed as means and standard deviations while qualitative variables as frequencies and percentages. For analysing the associations between proinflammatory variables with the different IL-6 groups, the chi-square test and analysis of variance (ANOVA) were implemented. The statistical methods for multivariate analysis were linear regression and logistic regression in order to compute the crude odds ratios and their confidence interval. Variables with a *p* value <0.05 in univariate models were selected in the multivariable tests. To reduce the extraction day bias and potential confounding variables, multivariable models were adjusted by age, sex, Charlson Comorbidity Index Score, main analytical values, and extraction day. Moreover, appropriate covariables were fitted to adjust the regression models. The program used for the statistical procedures was the STATA platform (version 12.1 for Windows, Texas, USA), whose manual and instructions were followed to perform the analyses. The statistical significance level was set at a two-sided *p* value of <0.05.

## 3. Results

The main clinical features of the cohort are summarised (Table 1). Out of 165 participants, 66% were men and 33% women. According to the IL-6 levels, there were 55 participants in each group. Forty-four percent presented arterial hypertension, 53% dyslipidaemia, and 33% diabetes. The average hospital stay days was 14 days, while 10% of the participants died in hospital wards and 13.3% required admission to the ICU.

The associations between serum IL-6 levels at admission and other proinflammatory variables are reported (Table 2). The relationships between IL-6 values and hospital stay days resulted statistically significant (*p* < 0.001), as well as ICU admission and mortality rate (*p* < 0.001). In addition, statistically significant differences in Charlson Comorbidity Index Score, ALT levels, CRP values, NLI, RDW, ferritin, and baseline blood glucose were found.

Coefficients for hospital stay days adjusted by mediators of inflammation in a multivariate model were calculated (Table 3). The association with age and IL-6 levels was statistically significant (*p* = 0.02 and *p* < 0.001, respectively). Coefficients were standardised so that the variances of dependent and independent variables are 1.

Associations between admission to ICU and the main proinflammatory variables based on logistic regression analyses are reported (Table 4). A statistically significant relationship was found between IL-6 levels and ICU admission (*p* = 0.006).

The odds ratio for mortality and the associated relevance of inflammatory biomarkers in a multivariate model are displayed (Table 5). Age, sex, NLI, ferritin, and serum levels of IL-6 showed a statistically significant association with in-hospital mortality (*p* < 0.05).

## 4. Discussion

In our analyses, data from 165 successively admitted patients with COVID-19 respiratory infection were studied, being selected in three groups according to clinically accepted IL-6 ranges. A multivariate model is presented with other possible laboratory indicators including relevant covariates. The analyses were performed following validated laboratory techniques at the Central Biochemistry Unit of the hospital, while suitable statistical methods were implemented as advised.

Serum cytokine levels have been previously described as important prognosis factors in other pandemic infectious outbreaks [18]. Given the interindividual differences among patients, precision pharmacological research and personalised medicine are becoming clinically relevant [9, 19], which demands the patient's categorisation for individualized anti-inflammatory management. Indeed, knowledge about inflammatory patterns for subsequent subject stratification may let the clinician to an earlier approach concerning the patient situation to prevent adverse outcomes and prescribe therapies according to specific pathological profiles [20]. Remarkable advantages of initial IL-6 assessment include to be a minimally invasive determination, be easily accessible, and be relatively inexpensive. Information provided by biomarker tools could contribute to understand the inflammatory condition and individually treat this novel disease with such an exponential growth [21].

A remarkable point of this study is the association between objectively measured IL-6 serum levels and poor prognosis determinants such as longer hospitalisation stays besides higher ICU admission and mortality rates. Multiple authors have reported that almost 80% of fatal cases exhibited above-normal circulating IL-6 values [22]. The so-called cytokine storm leads to T cell dysfunction and peripheral lymphopenia in most admitted patients and therefore evolving to severe forms of the disease [1]. In this context, anticytokine treatment has been widely demonstrated as useful in rheumatologic diseases (anakinra, tocilizumab, or baricitinib, for example), being administrated to infected patients all over the world with promising results [23] in an attempt to attenuate the host immune response to SARS-CoV-2 pathogen and associated inflammatory features. In fact, these therapies as well as glucocorticoids were included in our own centre protocol, with successful outcomes in hospitalised cases [7].

The cytokine storm resulting from the release of inflammatory mediators induces endothelial activation, capillary leak, circulatory collapse, clotting, and microvascular obstruction [11]. In addition, SARS-COV-2 triggers complex molecular events related to hyperinflammation with a putative role for IL-6, where several molecular axes associated to ACE-2 are involved [9].

In addition, there are evidences of a relationship between proinflammatory profiles and the personalised response to SARS-CoV-2 infection, which is also found in this cohort. Underlying diabetes (baseline hyperglycaemia) [24] and cardiovascular diseases and risks (hypertension, dyslipidaemia, smoking) worsen clinical outcomes to viral infections [25]. Indeed, serologic markers (IL-6 and CRP, for example) are elevated in patients with these inflammatory-related comorbidities linked to an endothelial damage, which is possibly due to potentiating “stormy” synergic influences. A meta-analysis showed a higher probability of sickness, development of severe symptoms, and fatal outcomes similar to those described according to MERS (Middle East Respiratory Syndrome) infection [26]. Furthermore, “inflammaging” [27] and obesity [28] are other conditions related with COVID-19 symptoms, prognosis, and adverse clinical consequences.

According to our results, IL-6 and the assessed inflammatory biomarkers followed analogous patterns. Thus, ferritin and the NLI showed statistically significant associations with mortality rates. Moreover, ARDS related to COVID-19 pneumonia generates a virally induced immunosuppression similar to primary haemophagocytic lymphohistocytosis [29]. This finding is a possible explanation of the shared analytical features concerning the macrophage activation-like syndrome such as hyperferritinaemia, abnormal hepatic profile, or coagulopathy. McGonagle et al. [30] showed that the macrophagic activation syndrome in SARS-COV-2 infection is related to nonsurvival in these subjects as well as IL-6 and IL-1 elevation. In addition, high viral load and continued exposure to the virus could be an important factor for the severity of disease [31]. Interestingly, the Charlson Comorbidity Index Score, transaminases, and CRP values exhibited the expected trends concerning inflammatory processes depending on circulating IL-6 levels [32].

In this context, NLI revealed a parallel trend with IL-6 and hospitalisation stage, ICU admission, and mortality rate in our series. NLI has emerged as an affordable inflammatory clinical marker with a predicting role in cancer, infectious diseases, cardiovascular complications, or autoimmune disease disorders [33, 34]. Necropsy data in SARS-COV-2-infected patients is low, but impaired myelopoiesis and lymphohematopoietic system involvement was noted within a hyperinflammation status, whose progression may have a high discrimination level in severe cases affecting inflammation mediators [35].

Some limitations cannot be denied. A cohort of 165 cases may not be representative of the whole population, which makes it convenient to validate these outcomes by further investigations. In any case, the achieved results are compatible with existing scientific evidences [36]. The absence of statistical significance in some biomarkers may be due to the relatively low number of subjects, but despite type 1 and type 2 statistical errors cannot be discarded, our results are clinically plausible. The extraction day of the blood samples may also induce a bias in some analyses, which was minimised with an adjusted multivariate model. Despite ferritin data are statistically significant, caution should be paid when interpreting such results given the small size magnitude found in this cohort. As for strengths, the homogeneity of the sample is a remarkable point due that the same protocol was followed by all clinicians in our hospital. In addition, the high sensitivity and specificity of IL-6 as the main serologic biomarker is another positive issue of the study. Indeed, the consideration of lifestyle factors (smoking and alcoholic drinking), body adiposity, and accompanying comorbidities in the inflammatory analyses [37, 38] adds great value to the current data since they were not usually screened in previous COVID-19 publications.

As a corollary, this research reproduces not only the validity of IL-6 as a prognostic COVID-19 factor but also demonstrated relevant associations and interactions with various inflammatory functions. These findings are in agreement with the suspected underlying physiopathological mechanisms induced by viral infections and providing additional information about other inflammatory contributors such as NLI, ferritin, and RDW that explain the assessed clinical outcomes and correlations with metabolically related comorbidities. Indeed, IL-6 measurements evidenced a worthy association with days of hospital stay days, ICU admission, and mortality rates with affordable value for personalised clinical practice and infection management.

## Figures and Tables

**Table 1:** Clinical features concerning the enrolled COVID-19 population.

Variables	Total (*n* = 165)
Age, years	62.79 (11.61)
Sex	
Male	109 (66.1)
Female	56 (33.9)
IL-6 levels	
0-10 pg/ml	55 (33.3)
10-100 pg/ml	55 (33.3)
>100 pg/ml	55 (33.3)
Days of symptomatology at admission	7.6 (4.0)
Hypertension	66 (44.0)
Dyslipidaemia	53 (32.1)
Diabetes mellitus	33 (20.0)
Charlson Comorbidity Index Score	0.84 (1.42)
ALT (U/l)	40.6 (29.9)
AST (U/l)	51.1 (30.4)
GGT (U/l)	86.7 (95.1)
LDH (U/l)	358.1 (139.4)
CRP (mg/l)	132.8 (143.7)
Leukocytes (counts/*μ*l)	7636 (3564)
NLI	7.9 (7.9)
RDW (%)	13.3 (1.2)
D-Dimer (*μ*g/ml)	1.71 (5.44)
Ferritin (ng/ml)	1065.3 (917.6)
Total cholesterol (mg/dl)	138.8 (34.3)
SBP (mmHg)	137.8 (21.9)
DBP (mmHg)	79.1 (14.1)
Baseline blood glucose (mg/dl)	130.2 (59.1)
BMI (kg/m^2^)	28.4 (4.6)
Current smoker	11 (7.01)
Standard drink units	3.69 (23.07)
Hospital stay days	14.0 (13.1)
ICU admission	22 (13.3)
Mortality	16 (10.1)

ALT: alanine aminotransferase; AST: aspartate aminotransferase; GGT: Gamma Glutamyl Transpeptidase; LDH: lactate dehydrogenase; CRP: C-reactive Protein; NLI: Neutrophil/Lymphocyte index; RDW: Red Cell Distribution Width; SBP: Systolic Blood Pressure; DBP: Diastolic Blood Pressure; BMI: Body Mass Index; SD: standard deviation. ^∗^Qualitative variables are expressed in number and proportion. Quantitative variables are expressed in mean and standard deviation.

**Table 2:** Association between IL-6 levels, clinical, anthropometrical, and biochemical determinants.

	Group 1 IL − 6 > 100 pg/ml, *N* = 55	Group 2 IL-6 10-100 pg/ml, *N* = 55	Group 3 IL − 6 < 10 pg/ml, *N* = 55	Chi^2^/ANOVA	*p*
Hypertension	26 (42.3)	25 (45.4)	15 (27.3)	5.61	0.06
Dyslipidemia	23 (41.8)	15 (27.3)	15 (27.3)	3.55	0.17
Diabetes mellitus	15 (27.3)	11 (20.0)	7 (12.7)	3.64	0.16
Charlson Comorbidity Index Score	1.33 (1.66)	0.55 (1.02)	0.64(1.38)	12.68	0.006
ALT (U/l)	47.1 (20.1)	40.8 (22.9)	49.2 (43.0)	57.39	0.01
AST (U/l)	32.0 (15.0)	53.9 (22.8)	52.3 (43.2)	37.93	0.48
GGT (U/l)	71.4 (76.7)	109.9 (121.1)	79.2 (79.7)	15.42	0.08
LDH (U/l)	350.1 (117.6)	388.5 (149.0)	335.3 (157.0)	4.17	0.14
CRP (mg/dl)	182.1 (217.8)	127.9 (70.7)	88.2 (72.2)	90.67	0.02
Leucocytes (counts/*μ*l)	7763 (4026)	7849.4 (3075)	7304 (3553)	3.76	0.69
NLI	10.9 (10.6)	7.2 (5.4)	5.6 (5.3)	34.56	0.001
RDW (%)	13.6 (1.3)	13.2 (1.2)	13.0 (0.9)	5.96	0.02
D-Dimer (*μ*g/ml)	1.60 (2.11)	2.40 (9.01)	1.05 (0.96)	205.80	0.44
Ferritin (ng/ml)	1283(953)	1096 (874)	806 (877)	0.41	0.05
Total cholesterol (mg/dl)	135.3 (33.2)	139.5 (25.2)	145.6 (36.6)	7.60	0.20
SBP (mmHg)	139.2 (24.2)	138.1(21.3)	136.2 (20.3)	1.82	0.78
DBP (mmHg)	78.6 (14.4)	79.4 (12.8)	79.2 (15.3)	1.73	0.94
Baseline blood glucose (mg/dl)	148.1 (77.0)	118.9(44.3)	120.9 (41.2)	24.49	0.01
BMI (kg/m^2^)	28.8 (4.5)	29.0 (5.0)	27.2 (3.9)	2.60	0.09
Current smoker	7 (12.7)	3 (5.4)	1 (1.8)	5.91	0.05
Drink standard units	8.76 (39.94)	1.74 (4.40)	0.78 (2.47)	1.87	0.15
NLI 2019	2.0 (3.5)	2.8 (4.2)	1.7 (0.8)	59.75	0.28
Hospital stay days	22.0 (17.1)	11.9 (10.4)	8.6 (5.8)	18.04	<0.001
ICU admission	16 (29.1)	4 (7.2)	2 (3.6)	18.04	<0.001
Mortality	13 (23.6)	3 (5.4)	0 (0.0)	20.67	<0.001

ALT: alanine aminotransferase; AST: aspartate aminotransferase; GGT: Gamma Glutamyl Transpeptidase; LDH: lactate dehydrogenase; CRP: C-reactive Protein; NLI: Neutrophil/Lymphocyte index; RDW: Red Cell Distribution Width; SBP: Systolic Blood Pressure; DBP: Diastolic Blood Pressure; BMI: Body Mass Index. ^∗^Qualitative variables are expressed in number and proportion. Quantitative variables are expressed in mean and standard deviation.

**Table 3:** Coefficients for hospital stay days adjusted by age, sex, Charlson Comorbidity Index Score, and main analytical values in a linear regression multivariate model.

Hospital stay days	Coefficient	95% conf. interval	*p* value
Sex	-0.86	(-5.36, 3.63)	0.70
Age	-0.24	(-0.45, -0.04)	0.02
Charlson Comorbidity Index Score	0.53	(-0.88, 1.96)	0.46
CRP (mg/dl)	-0.01	(-0.04, 0.004)	0.11
NLI	0.13	(-0.19, 0.47)	0.40
RDW (%)	-0.37	(-2.32, 1.56)	0.70
Ferritin (ng/ml)	0.001	(-0.001, 0.003)	0.34
Extraction day	-0.41	(-1.78, 0.94)	0.55
IL-6 (pg/ml)	6.78	(9.60, 3.96)	<0.001

LDH: lactate dehydrogenase; CRP: C-reactive Protein; NLI: Neutrophil/Lymphocyte index; RDW: Red Cell Distribution Width; IL-6: Interleukin 6. ^∗^LDH was excluded because of collinearity.

**Table 4:** Odds ratio (OR) for UCI admission adjusted by age, sex, Charlson Comorbidity Index Score, and main analytical values in a multivariate model.

ICU admission	Odds ratio	95% conf. interval	*p* value
Sex	1.55	(0.25, 9.71)	0.64
Age	0.93	(0.86, 1.00)	0.06
Charlson Comorbidity Index Score	0.56	(0.26, 1.22)	0.14
LDH (U/l)	0.99	(0.98, 1.00)	0.06
CRP (mg/dl)	1.00	(0.99, 1.00)	0.64
NLI	1.08	(0.99, 1.17)	0.07
RDW (%)	1.07	(0.43, 2.63)	0.89
Ferritin (ng/ml)	1.00	(0.99, 1.00)	0.40
Extraction day	0.67	(0.40, 1.12)	0.12
IL-6 (pg/ml)	24.93	(2.49, 249.63)	0.006

LDH: lactate dehydrogenase; CRP: C-reactive Protein; NLI: Neutrophil/Lymphocyte index; RDW: Red Cell Distribution Width; IL-6: Interleukin 6.

**Table 5:** Odds ratio (OR) for in-hospital mortality adjusted by age, sex, Charlson Comorbidity Index Score, and main analytical values in a multivariate model.

Mortality	Odds ratio	95% conf. interval	*p* value
Sex	10.12	(1.09, 94.18)	0.04
Age	1.19	(1.05, 1.34)	0.007
Charlson Comorbidity Index Score	1.30	(0.76, 2.22)	0.33
LDH (U/l)	0.99	(0.98, 1.00)	0.23
CRP (mg/dl)	1.00	(0.99, 1.01)	0.84
NLI	1.20	(1.02, 1.42)	0.03
RDW (%)	1.20	(0.66, 2.18)	0.55
Ferritin (ng/ml)	1.00	(1.002, 1.003)	0.02
Extraction day	0.74	(0.36, 1.52)	0.41
IL-6 (pg/ml)	9.81	(1.56, 61.69)	0.02

LDH: lactate dehydrogenase; CRP: C-reactive Protein; NLI: Neutrophil/Lymphocyte index; RDW: Red Cell Distribution Width; IL-6: Interleukin 6.

## Data Availability

The data of this research are deposited in the database of the Internal Medicine Department, Hospital Puerta de Hierro Majadahonda. The authors will provide information if they receive reasonable requests.

## Abbreviations

ALT: Alanine aminotransferase
AST: Aspartate aminotransferase
BMI: Body Mass Index
CRP: C-reactive Protein
DBP: Diastolic Blood Pressure
GGT: Gamma Glutamyl Transpeptidase
IL-6: Interleukin 6
LDH: Lactate dehydrogenase
NLI: Neutrophil/Lymphocyte index
RDW: Red Cell Distribution Width
SBP: Systolic Blood Pressure.
